# Fireworms are a reservoir and potential vector for coral-infecting apicomplexans

**DOI:** 10.1093/ismejo/wraf078

**Published:** 2025-04-24

**Authors:** Anthony M Bonacolta, Bradley A Weiler, Candace J Grimes, Morelia Trznadel, Mark J A Vermeij, Patrick J Keeling, Javier del Campo

**Affiliations:** Department of Botany, University of British Columbia, 3529-6270 University Boulevard, Vancouver, BC V6T 1Z4, Canada; Department of Marine Biology and Ecology, Rosenstiel School of Marine, Atmospheric, and Earth Science, University of Miami, 4600 Rickenbacker Cswy, Miami, FL 33149, United States; Institut de Biologia Evolutiva (CSIC-Universitat Pompeu Fabra), Passeig Marítim de la Barceloneta 37-49, Barcelona, Catalonia 08003, Spain; Department of Marine Biology and Ecology, Rosenstiel School of Marine, Atmospheric, and Earth Science, University of Miami, 4600 Rickenbacker Cswy, Miami, FL 33149, United States; Institut de Biologia Evolutiva (CSIC-Universitat Pompeu Fabra), Passeig Marítim de la Barceloneta 37-49, Barcelona, Catalonia 08003, Spain; Marine Biology Department, Texas A&M University of Galveston, 200 Seawolf Parkway, Galveston, TX 77554, United States; Department of Botany, University of British Columbia, 3529-6270 University Boulevard, Vancouver, BC V6T 1Z4, Canada; CARMABI Foundation, Piscaderabaai z/n, P.O. Box 2090, 42CJ+WH Willemstad, Curaçao; Department of Freshwater and Marine Ecology, Institute for Biodiversity and Ecosystem Dynamics, University of Amsterdam, P.O. Box 94240, Amsterdam 1090 GE, The Netherlands; Department of Botany, University of British Columbia, 3529-6270 University Boulevard, Vancouver, BC V6T 1Z4, Canada; Department of Botany, University of British Columbia, 3529-6270 University Boulevard, Vancouver, BC V6T 1Z4, Canada; Department of Marine Biology and Ecology, Rosenstiel School of Marine, Atmospheric, and Earth Science, University of Miami, 4600 Rickenbacker Cswy, Miami, FL 33149, United States; Institut de Biologia Evolutiva (CSIC-Universitat Pompeu Fabra), Passeig Marítim de la Barceloneta 37-49, Barcelona, Catalonia 08003, Spain

**Keywords:** parasitism, vectors, microbiome, coral reefs, holobionts, *Apicomplexa*, polychaetes, corals, symbiosis

## Abstract

Corals (*Cnidaria*; *Anthozoa*) play critical roles as habitat-forming species with a wide range, from warm shallow-water tropical coral reefs to cold-water ecosystems. They also represent a complex ecosystem as intricate holobionts made up of microbes from all domains of the Tree of Life that can play significant roles in host health and fitness. The corallicolids are a clade of apicomplexans that infect a wide variety of anthozoans worldwide and can influence the thermal tolerance of habitat-forming corals. Despite their potentially important impacts on reef ecosystems, much of the basic biology and ecology of corallicolids remains unclear. Apicomplexans often have a closed life cycle, with minimal environmental exposure and sometimes multiple hosts. Corallicolids have only been documented in anthozoan hosts, with no known secondary/reservoir hosts or vectors. Here, we show that abundant corallicolid sequences are recovered from bearded fireworms (*Hermodice carunculata*) in tropical reef habitats off Curaçao and that they are distinct from corallicolids infecting the corals on which the fireworms were feeding at the time of their collection. These data are consistent with a fireworm-specific corallicolid infection, not merely a byproduct of the worms feeding on infected corals. Furthermore, we suggest that *H. carunculata* is potentially a vector moving corallicolids among coral hosts through its feces. These findings not only expand our understanding of the ecological interactions within coral reef ecosystems but also highlight the potential role of host-associated parasites in shaping the resilience of reef habitats.

## Introduction

**Figure 1:**
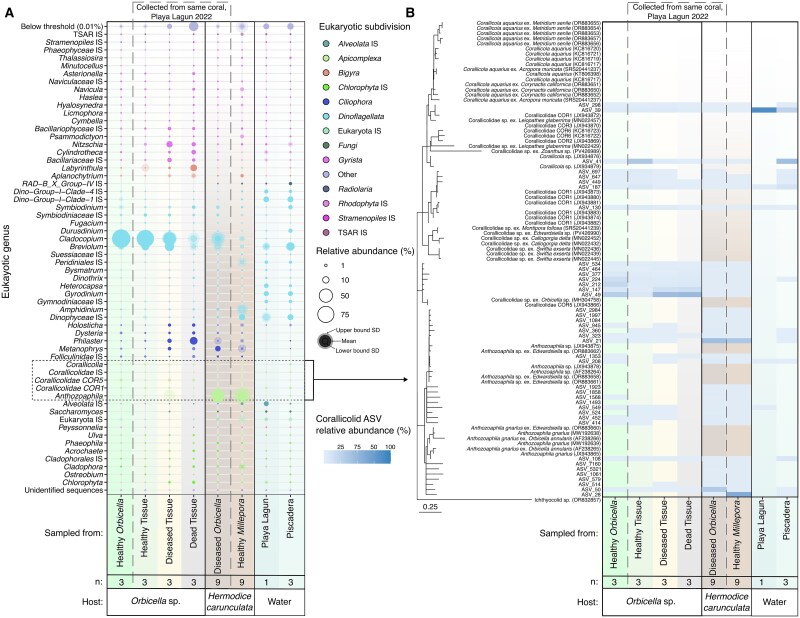
**18S rRNA gene amplicon sequencing.** (A) Relative abundance bubble plot of 18S rRNA genes across *O. annularis*, *H. carunculata*, and water samples. Bubble shading corresponds to the standard deviation of the samples within each category. The dashed gray box indicates the coral and worm samples taken from the same coral colony in Playa Lagun, Curaçao, in October 2022. The sample number per category is represented by “n”. (B) ASVs classified as corallicolids were EPA-placed onto a backbone 18S–28S *rrn* operon tree of *Corallicolida*. Tree scale is 0.25. Ichthyocolids were used as the outgroup. The relative abundance of each ASV is shown in the heat-map across the same sample categories from panel A.

Multiple *Hermodice carunculata* individuals were observed feeding on the diseased tissue (black band disease) of the boulder star coral, *Orbicella annularis*, at Playa Lagun in western Curaçao (Supplementary Fig. 1). Triplicate samples were taken from the following: three individual worms, coral tissues from a disease transect of *O. annularis* (apparently healthy tissue, diseased transition line tissue, and subsequent dead tissue; Supplementary Fig. 1), a proximally adjacent healthy *O. annularis* individual, three fireworms taken from around a healthy *Millepora* sp. colony on a different reef near Piscadera in central Curaçao, and individual water samples from both sampling locations (taken at sampling depth, 1 L water filtered through a 0.2 μm filter). Samples were stored in Zymo DNA/RNA shield first at room temperature during travel then at −20°C in the lab before being extracted with the Zymobiomics DNA/RNA Miniprep kit (Catalogue R2002). DNA extracts were PCR-amplified for the V4 regions of 18S rRNA and 16S rRNA genes using established protocols to investigate the eukaryotic nuclear and plastid gene abundances, respectively [1, 2]. For this purpose, the 16S rRNA gene dataset was filtered to include only plastid amplicon sequence variants (ASVs). Corallicolids [3, 4], specifically *Anthozoaphila* spp., were found to be the primary microeukaryote recovered from *H. carunculata*, often making up over 50% of the reads (Fig. 1A). Conversely, corals were primarily dominated by *Symbiodiniaceae*, which gradually decreased in abundance across the disease transect (towards dead tissue). Corallicolids, even though they still make up a portion of the microeukaryotic community, were not detected in high abundance across the sampled corals nor in the water in which they were found (Fig. 1A).

The primary corallicolid ASVs from all samples were placed using RAxML’s Evolutionary Placement Algorithm (EPA) [5] onto an *rrn* operon (18S + 28S rRNA genes) backbone reference tree to investigate the relatedness of recovered sequences. The predominant corallicolid ASVs from *H. carunculata* were found to branch with *Anthozoaphila gnarlus*, distinctly distant from sequences acquired from the coral on which they were feeding: the coral-derived corallicolids branched with a clade previously recovered from another *O. annularis* (Fig. 1B). The recovery of distinct *A. gnarlus-*related ASVs from multiple *H. carunculata* individuals from multiple locations is notable, as is the absence of corallicolid reads in *H. carunculata* that match those of the disease lesion of the *O. annularis* colony where the worms were feeding. Although corallicolids comprised a tiny portion of the microeukaryotic community in water samples, the predominant corallicolid ASV was again distinct from both the coral and fireworm-associated clades, branching with *Corallicolidae COR1* (Fig. 1B).

**Figure 2:**
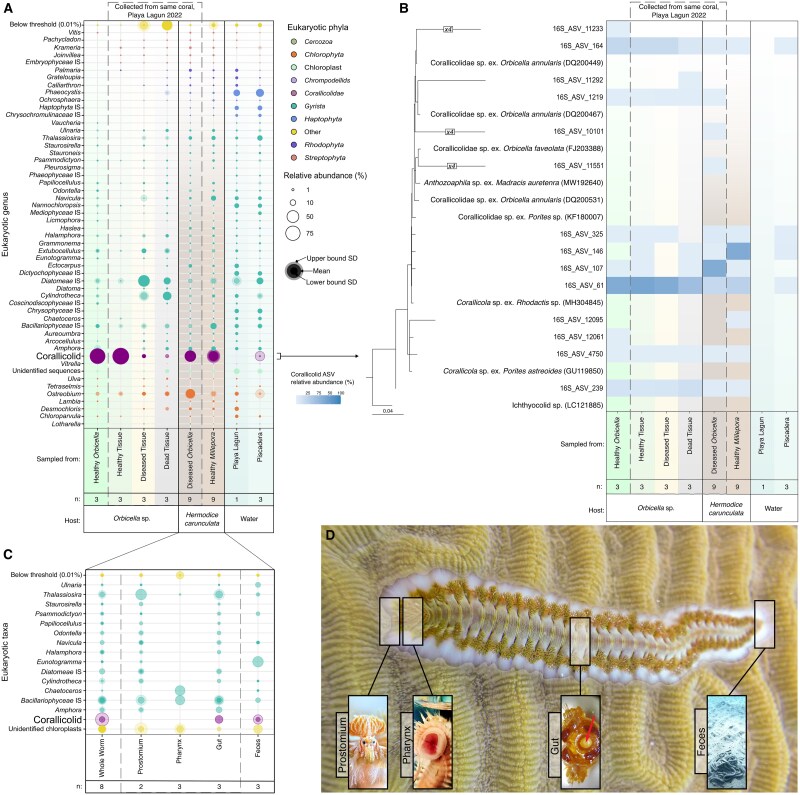
**Plastid 16S rRNA gene amplicon sequencing.** (A) Relative abundance bubble plot of plastid 16S rRNA genes across *O. annularis*, *H. carunculata*, and water samples. Bubble shading corresponds to the standard deviation of the samples within each category. The dashed gray box indicates the coral and worm samples taken from the same coral colony in Playa Lagun, Curaçao, in October 2022. The sample number per category is represented by “n”. (**B**) Plastid 16S rRNA gene ASVs confirmed as corallicolids were EPA-placed onto a backbone 16S rRNA gene tree of *Apicomplexa* (only the corallicolid portion is shown). Tree scale is 0.04. The relative abundance of each plastid ASV is shown in the heat-map across the same sample categories from panel A. (**C**) Relative abundance bubble plot of plastid 16S rRNA genes across fire worm sampling sites, indicating the presence of corallicolid ASVs in the whole worm, gut, and feces samples. The sample number per category is represented by “n”. (**D**) Diagram of *H. carunculata* sampling strategy. Close-up images courtesy of Gabriel Jensen and Candace Grimes.

To better compare our results with other datasets, the plastid 16S rRNA gene was also acquired, as it is a much more common molecular marker in environmental surveys. These data confirmed the high abundance of corallicolids in all *H. carunculata* samples based on plastid 16S rRNA gene abundance (42.1 ± 1.65% in *Orbicella* & 46.7 ± 13.2% in *Millepora*; Fig. 2A). We also again recovered different dominant plastid ASVs for coral, fireworms, and water, respectively. All corallicolid ASVs branch together on an EPA-placement tree of previously recovered corallicolid plastid 16S rRNA genes (Fig. 2B), unlike the corallicolid nuclear 18S rRNA genes (Fig. 1B). This may be explained by the substitution rate of 18S rRNA gene being much higher than that of the 16S rRNA gene, and the plastid gene accordingly failing to resolve closely related lineages.

To examine the tissue specificity in *H. carunculata*, we analyzed 16S rRNA genes from whole worms and from multiple tissues (prostomium, pharynx, gut, and feces) in dissected *H. carunculata* from Curaçao in 2018. Corallicolid plastid 16S rRNA genes were also abundant in the whole worms and the gut and feces but absent in other tissues (Fig. 2C).

The data presented here provides the first evidence for a non-anthozoan host of corallicolids. Indeed, the abundance of corallicolids within *H. carunculata* is high compared to the coral specimen. These data also lead to questions of whether *H. carunculata* is infected with a distinct subtype of corallicolid or whether the lifecycle of coral-infecting corallicolids may include other hosts, such as *H. carunculata.* Corallicolids are related to coccidians [6], and many coccidians alternate between sexual reproduction within a primary host and asexual reproduction in a secondary host. The closest sister group to corallicolids is the ichthyocolids, which are blood parasites of marine fishes that are obligately transmitted by gnathiid vectors [7, 8]. It is altogether not unlikely, therefore, that corallicolids also have a life cycle that alternates between hosts. Fireworms may play a key role in the potentially complex life cycle of corallicolids, but further research is needed to clarify the different life stages in fireworms and coral.

Since the dominant corallicolids recovered from the diseased coral were distinct from those of the fireworms actively feeding on the coral, we can conclude that the corallicolids within the fireworms did not originate from that feeding. Therefore, the *Anthozoaphila* spp*.* from the fireworms may have either originated from a prior coral host with which the fireworm has interacted or represent a corallicolid lineage with an especially wide host range, including both corals and fireworms. In the first case, we see that the *Anthozoaphila* spp. within the fireworms are highly abundant within the worms at the time of sampling and are therefore capable of being vectorized to a coral not yet highly infected with this lineage.

These data suggest corallicolid transmission may be a complex mix of several factors, potentially different in different parasite lineages. For example, brooding coral species have been proposed to transmit corallicolids vertically [9]. Still, another transmission mode is required to explain infection in broadcast-spawning coral species, such as *Orbicella* spp. Fireworms as a vector might fill this gap, as they are common generalist corallivores in tropical reef ecosystems [10], and a fecal-oral transmission mode is consistent with the data. How dynamic this picture might be is exemplified when one also considers that fireworms are invasive in some regions, like the Mediterranean Sea [11]. Also, fireworms have the potential to expand to other ocean regions, such as the Indo-Pacific through the Suez Canal, as a result of the climate crisis and the tropicalization of the Mediterranean Sea [12], so their role as vectors could impact the health of reefs in these regions [13, 14]. And if fireworms are indeed vectors, they cannot be the only species in this role because corallicolids are found in Anthozoans from the deep sea and northern latitudes [15, 16], both outside the range of *H. carunculata* [17], so other, un-explored vectors may operate in these ecosystems. To confirm fireworms or any other organism as a corallicolid vector, future research should investigate whether corallicolids can tolerate passage through the vector and be effectively taken up by a coral. Future research should also focus on isolating and sequencing the corallicolids from *H. carunculata* to clarify their association with this host and to expand the search to other potential non-anthozoan hosts to clarify their ecological link to infected anthozoans. Understanding corallicolid ecology in the anthozoan holobiont is critical to uncovering these parasites' influence in the unprecedented environmental changes these ecosystems are experiencing.

## Supplementary Material

disease_transect_wraf078

## Data Availability

The raw reads for the project have been deposited on NCBI SRA (BioProject: PRJNA1222990). Code used for analysis and ASV count tables, taxonomy tables, and nucleotide sequences can be found on GitHub at: https://github.com/delCampoLab/Hermodice_corallicolids/.
