# Supplementary figures and images for: Fireworms are a reservoir and potential vector for coral-infecting apicomplexans
Source: ISME J. 2025 Apr 24;19(1):wraf078. doi: 10.1093/ismejo/wraf078 (PMC12054993; doi:10.1093/ismejo/wraf078)

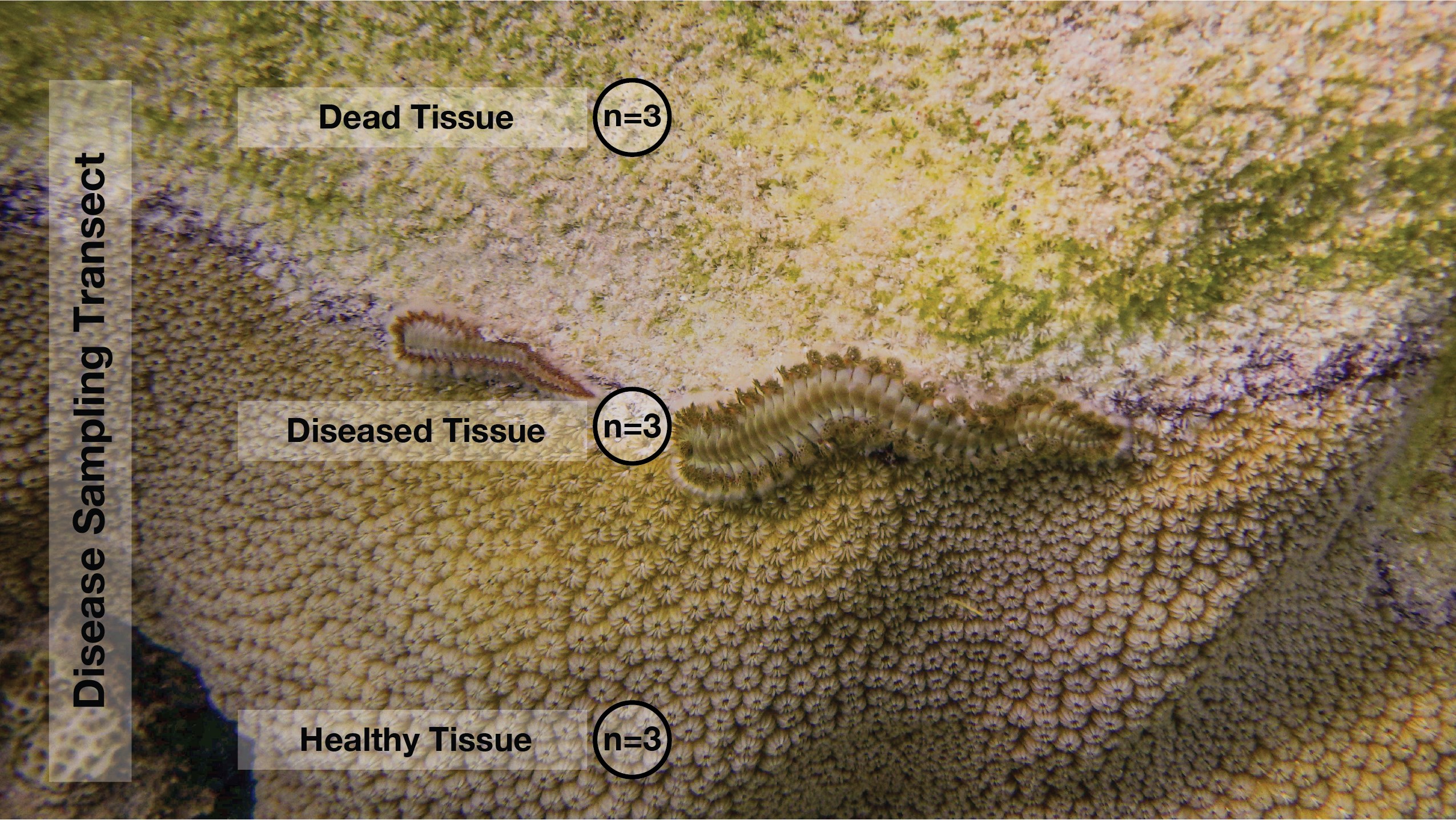

Supplement: disease_transect_wraf078 [file disease_transect_wraf078.jpeg]
